# Evaluating dosimetric accuracy of the 6 MV calibration on EBT3 film in the use of Ir‐192 high dose rate brachytherapy

**DOI:** 10.1002/acm2.13571

**Published:** 2022-02-28

**Authors:** Lyu Huang, Hani Gaballa, Jenghwa Chang

**Affiliations:** ^1^ Department of Radiation Medicine Center for Advanced Medicine Northwell Health New Hyde Park New York USA; ^2^ Department of Radiation Medicine Donald and Barbara Zucker School of Medicine at Hofstra/Northwell New Hyde Park New York USA; ^3^ Department of Physics and Astronomy Hofstra University Hempstead New York USA

**Keywords:** EBT3 film, film calibration, HDR brachytherapy, radiochromic film dosimetry

## Abstract

**Purpose:**

To evaluate the dosimetric accuracy of EBT3 film calibrated with a 6 MV beam for high dose rate brachytherapy and propose a novel method for direct film calibration with an Ir‐192 source.

**Methods:**

The 6 MV calibration was performed in water on a linear accelerator (linac). The Ir‐192 calibration was accomplished by irradiating the film wrapped around a cylinder applicator with an Ir‐192 source. All films were scanned 1‐day post‐irradiation to acquire calibration curves for all three (red, blue, and green) channels. The Ir‐192 calibration films were also used for single‐dose comparison. Moreover, an independent test film under a H.A.M. applicator was irradiated and the 2D dose distribution was obtained separately for each calibration using the red channel data. Gamma analysis and point‐by‐point profile comparison were performed to evaluate the performance of both calibrations. The uncertainty budget for each calibration system was analyzed.

**Results:**

The red channel had the best performance for both calibration systems in the single‐dose comparison. We found a significant 4.89% difference from the reference for doses <250 cGy using the 6 MV calibration, while the difference was only 0.87% for doses >600 cGy. Gamma analysis of the 2D dose distribution showed the Ir‐192 calibration had a higher passing rate of 91.9% for the 1 mm/2% criterion, compared to 83.5% for the 6 MV calibration. Most failing points were in the low‐dose region (<200 cGy). The point‐by‐point profile comparison reported a discrepancy of 2%–3.6% between the Ir‐192 and 6 MV calibrations in this low‐dose region. The linac‐ and Ir‐192‐based dosimetry systems had an uncertainty of 4.1% (*k* = 2) and 5.66% (*k* = 2), respectively.

**Conclusions:**

Direct calibration of EBT3 films with an Ir‐192 source is feasible and reliable, while the dosimetric accuracy of 6 MV calibration depends on the dose range. The Ir‐192 calibration should be used when the measurement dose range is below 250 cGy.

## 1 INTRODUCTION

The radiochromic film has replaced its predecessor, the radiographic film, as the film of choice for clinical radiology/radiotherapy applications since it eliminates the need for post‐processing and exhibits a wider dynamic range (up to hundreds of Gy). Therapeutic applications of radiochromic films include the patient‐specific Quality Assurance (QA),^1^, ^2^, ^3^, ^4^ linac commissioning,^5^ brachytherapy QA,^6^, ^7^, ^8^, ^9^, ^10^ treatment planning system (TPS) dose validation,^11^ and so forth. The major advantage of radiochromic film over other dosimeters in brachytherapy applications is its high spatial resolution, customizability into any shape, and 2D measurement capability. The widely used EBT3 radiochromic film is a relatively new generation of the EBT family from Ashland Inc. (Bridgewater, NJ) with a useful dose range up to 20 Gy. Its active layer (with a 1.6% Al additive in comparison to the previous generations) is sandwiched between two clear polyester layers with a total thickness of ∼278μm.^12^ After exposure, the photopolymerization process continues for 24 h,^13^, ^14^ during which the color of the film gradually turns dark blue.

Patient‐specific QA is a well‐accepted practice for Intensity‐Modulated Radiation Therapy (IMRT) plans in external beam radiotherapy.^15^ However, for brachytherapy, it is not common to verify the dose (distribution) with measurements before treatment even though a very high and localized dose is delivered. The argument is that the commercial applicators are well‐designed and usually have a very simple geometry. That is, there is a minimal chance of incorrect dose calculation. However, with the growing use of customized 3D‐printed applicators for achieving a highly conformed dose distribution in complex anatomy, validation of the dose distribution before treatment has become increasingly important. Rooney et al.^16^ reviewed the current practice of 3D‐printing technique in radiation oncology and concluded that brachytherapy applicator accounts for 20% of these applications. A study from Ricotti et al.^17^ also demonstrated that the infill percentage has a significant impact on the dose distribution of 3D‐printed flat applicators. For example, the gamma passing rate at 2 mm/2% gamma criteria was 83.6% for 40% infill percentage but increased to 94.5% when the infill percentage was reduced to 10%. EBT3 film has been a popular choice for validating the dose distribution of these 3D‐printed applicators.^18^, ^19^

A 6 MV photon beam is frequently used for film calibration in radiotherapy applications. However, most films suffer from a strong energy‐dependent response, particularly at energies much lower than 6 MV, that is, in the kV range. Various studies have been reported on the energy response of EBT3 film in the kV range. Massillon et al.^20^ reported a weak energy dependence between the 6 and 15 MV beams but a variation of more than 11% for lower energy photons (e.g., 50 kV) at a dose less than 2 Gy. Nevertheless, Brown et al.^21^ studied three monochromatic (25, 30, and 35 keV) photon beams produced by a synchrotron and unexpectedly found that the EBT3 film has a weak energy dependence (sensitivity between 0.97 and 0.99) compared to the 4 MV beam of a clinical radiotherapy accelerator. Later, Bekerat et al.^22^ investigated the energy dependence of different EBT, EBT2, and EBT3 film models for energies < 100 keV and its correlation with the active layer composition. They found that the latest commercial EBT3 film model with a 7% Al additive has an under‐response at all energies <100 keV, ranging from −6%±4% at 40 keV to −20%±4% at 20 keV. At the same time, Villarreal‐Barajas and Khan^23^ also reported a 5% under‐response in EBT3 film at 300 kVp and 20% at 70 kVp.

Ir‐192 decay has a complex gamma‐ray spectrum, ranging from 136 keV to 1.06 MeV, with an average energy of 380 keV. To avoid the energy response issue, several papers were published to calibrate the film directly using the Ir‐192 source with a parallel‐opposed beam geometry.^24^, ^25^, ^26^ Chiu‐Tsao et al.^24^ placed an EBT film piece below and above the Ir‐192 source at a distance of about 1.1 cm for film calibration, and calculated the dose to water at the film center with the TG‐43 formalism. They found the dose‐response of EBT film is nearly independent of the radiation energy between the Ir‐192 and 6 MV photon sources. Aldelaijan et al.^25^ placed a piece of EBT2 film between two catheters and calculated the dose using the MasterPlan TPS. They found superior precision with the green channel in dose range up to 50 Gy but had not compared the energy response between the two energy sources. Most of these direct calibration methods are difficult to implement due to uncertainties in source‐to‐film positioning and a longer irradiation time for a wider uniform dose strip at a farther distance. In the present study, we proposed a simple and effective method for direct film calibration with the Ir‐192 source using a cylinder applicator to compare the performance of EBT3 film calibration using the Ir‐192 and 6 MV photon sources.

The main goal of this work was to investigate the accuracy of the 6 MV calibration for the EBT3 film when it is used for the dosimetry of Ir‐192 high dose rate (HDR) brachytherapy. We have designed an innovative method for direct calibration of radiochromic films using the Ir‐192 source of the HDR unit at our institution, for the dose range of up 1000 cGy. This method allows in‐water calibration so that a uniform dose can be delivered on the film. Calibration with the 6 MV photon beam was also performed on a TrueBeam of our institution. The two calibrations were compared in terms of accuracy in both point‐dose measurement and 2D dose profile determination.

## 2 MATERIALS AND METHODS

### 2.1 6 MV calibration

**FIGURE 1:**
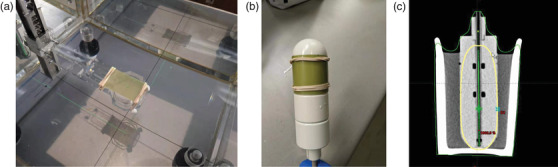
Film set‐up for the 6 MV and Ir‐192 calibrations. (a) Shows the film set‐up for the 6 MV calibration. The precut film was fixed on top of a Roos chamber holder attached to an MP1 phantom tank. (b) and (c) Illustrate the Ir‐192 calibration. The precut film piece was wrapped around and fixed to a cylinder high dose rate (HDR) applicator of 40 mm in diameter. (c) The setup was scanned, and a treatment plan was prepared for calibration with the Ir‐192 source

The 6 MV calibration of EBT3 film was performed using the 6 MV photon beam of a TrueBeam (Varian Medical Sys. Inc., Palo Alto, CA) at our institution with a dose rate of 400 MU/min. The dose range of this calibration was between 0 and 10 Gy, with 18 selected calibration dose levels in between (0, 25, 50, 75, 100, 125, 150, 200, 250, 300, 350, 400, 450, 500, 550, 600, 800, and 1000 cGy). Before performing the 6 MV calibration, the output of the TrueBeam was measured using a NIST‐traceable ionization chamber (PTW TN30013) in a PTW MP1 Water Phantom Tank (PTW‐Freiburg, Germany). The measured output, 1.004 cGy/MU combined with the TMR(10) and output factor of the 20 × 20 cm^2^ field size, was then used to calculate the MU for delivering each calibration dose level. We chose to irradiate the film inside the same water tank instead of a more convenient solid phantom because a direct measurement in water would eliminate the need for additional dose conversion from solid phantom to water. One sheet of EBT3 film (lot # 08032005) was first cut into 3.5 × 6 cm^2^ pieces with a paper cutter. A Roos chamber holder (PTW‐Freiburg) was used to hold the film because it has a wider opening, is made of water‐equivalent materials, and, therefore, would introduce less perturbation to the dose measurements. Before the experiment, the water tank and chamber holder were leveled to ensure the perpendicular beam incidence on the film. For each measurement, a piece of film was fixed to the top of the chamber holder with its longer side along with the chamber handle (Figure 1a) and aligned with the central axis of the 20 × 20 cm^2^ field. The film was then moved to the level of the water surface. After the position sensor was zeroed, the film was moved down to a depth of 10 cm, with at least 5 cm of water beyond for backscatter.

### 2.2 Ir‐192 calibration

The Ir‐192 calibration was performed by irradiating the film wrapped around a cylinder applicator of 40 mm in diameter as shown in Figure 1b, using the GammaMedplus iX afterloader (Varian Medical Sys. Inc.) of our institution. The 40‐mm cylinder applicator was chosen for this calibration because it is the largest size applicator available from the vendor. A larger diameter means a smaller curvature on the surface, or less likely to cause damage due to bending when a piece of film is wrapped around the applicator. The source strength was 5.958 Ci on the measurement day. The calibration was performed for 18 similar dose levels (0, 24, 50, 75, 101, 125, 150, 201, 251, 303, 351, 399, 449, 499, 549, 601, 802, and 1000.3 cGy) to that for the 6 MV calibration.

An EBT3 film (lot # 08032005) was cut into 4 × 6 cm^2^ film pieces with a paper cutter. For each film irradiation, a precut film piece was tightly wrapped around the surface of the cylinder applicator and fixed with two rubber bands (Figure 1b). Given the sharp dose fall‐off of the Ir‐192 source, this film setup was examined carefully before every measurement to avoid any air gaps that might significantly affect the accuracy of dose delivery. The cylinder applicator was then inserted into a water‐filled mug large enough to accommodate the whole cylinder applicator with at least two segments immersed in water. The cylinder applicator was immobilized using a lid made of a very thin piece of plastic with an opening at the center to allow the base of the applicator tube to pass. The whole setup was scanned in our CT simulator (SOMATOM Definition AS, Siemens Healthineers, Erlangen, Germany) using the abdomen protocol with a slice thickness of 0.6 mm. The CT images were exported to the Eclipse 15.6 TPS and the BrachyVision module was used to develop a treatment plan for each dose level. The user origin was set at the center of the cylinder and 4 cm away from the tip of the applicator. The source positions were selected to be equally spaced with a step size of 0.3 cm in the range of 0–10 cm. The dose distribution was optimized with the “Dose Shaper” tool to ensure the 100% isodose line touches the surface of the applicator. In this study, we aimed to have a uniform dose distribution on the applicator surface wrapped by the film, as illustrated in Figure 1c. To minimize the uncertainty brought by “Dose Shaper” in the calibration system, the radius of 100% isodose line, which should be 2 cm, was checked on axial slices in the area of interest. The dose calculation was done using the Acuros BV 1.7 algorithm with inhomogeneity correction and the dose was reported in the water. A dose reporting point located at 2 cm lateral to the user origin was created and considered as the reference dose, $D_{ref}$, delivered to the film.

### 2.3 Scanning protocol

**FIGURE 2:**
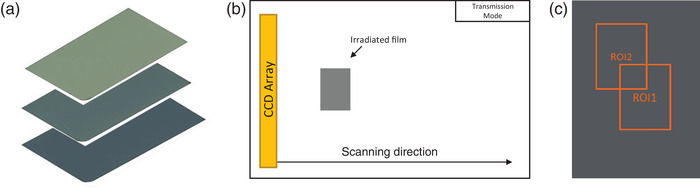
Illustration of the scanning protocol. (a) Example of an irradiated film set for calibration; (b) each film was sequentially positioned on the scanner bed and scanned; (c) different regions of interest (ROIs) are placed for calibration and verification purposes in RIT V6.8 Film package

Figure 2 illustrates the scanning protocol. For film scanning, we used an EPSON Expression 10000XL Wide‐Format Graphic Arts Scanner, a widely used flat‐bed scanner for film dosimetry. A customized film placement guide was constructed to position the film at the same position in the central area of the scanner each time to avoid the light scattering effect and improve repeatability. The film was scanned in the transmission mode with the reflective document mat removed. The scanning parameters were 48 bits, 150 dpi (or 0.168 mm per pixel), and TIFF format with no compression. Color correction was disabled to preserve the raw information. Each time before using, the scanner was warmed up with 2–3 unused scans.

Films were handled carefully when placed on the paper cutter or scanner bed to avoid any scratches, and gloves were worn all the time in order not to add any grease or fingerprints on the film. All irradiated films were scanned 24 h after exposure to allow time for polymerization. Each film piece was scanned in the “portrait” orientation. This was achieved by marking the direction of the longer side of the EBT3 film sheet on every film piece with an arrow. When a film piece was placed on the scanner bed, we made sure that the arrow pointed toward the moving direction of the scanner light source.

The RIT V6.8 Film software package, a commercial software developed by radiological imaging technology (RIT), Inc. for dosimetry and radiotherapy QA, was used in this study to generate calibration curves, convert pixel value to dose, and perform gamma analysis. The scanned image files in TIFF format from the two calibration sets were imported using the “perpendicular calibration” function with a region of interest (ROI) of 3 × 2 cm^2^. The ROI was placed at the center of the film image, and its mean pixel value was then associated with the dose delivered. All three (red, green, and blue) channels were scanned, analyzed, and used to generate the calibration curves of corresponding colors. Each calibration curve was applied to the verification and test films with the piecewise polynomial interpolation as recommended by RIT.

### 2.4 Single‐dose comparison

The film set for Ir‐192 calibration was also used as a verification set. An ROI (e.g., ROI_2_ in Figure 2c) different from those used in the calibration process (e.g., ROI_1_ in Figure 2c) was placed on these film pieces for all three channels, and the average dose to this ROI, ${\bar{D}}_{ROI}$ was determined using the 6 MV calibration curve. The percent error, *R*, between the average dose (${\bar{D}}_{ROI}$) and reference dose ($D_{ref}$) was estimated with Equation (1):

(1) $$R=\mathrm{abs}\left(\frac{{\bar{D}}_{ROI}-D_{ref}}{D_{ref}}\right)\times 100\%.$$

The analysis was repeated three times using three different ROIs. For comparison, the same analysis was also performed using the Ir‐192 calibration with the same ROIs.

### 2.5 Comparison of 2D dose distribution

**FIGURE 3:**
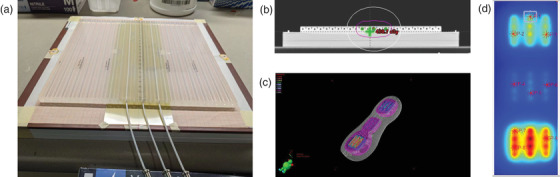
(a) Three radiopaque markers were placed on the phantom edge and a 7 × 25.4 cm^2^ film piece was placed in the central area under the H.A.M. applicator with three catheters inserted; (b) computed tomography (CT) scan of the measurement setup; (c) a peanut‐shaped dose distribution with high doses in its two ends and low dose in the middle was generated in Eclipse 15.6 TPS, which has three dose levels. The 100% isodose line represents 500 cGy. (d) Registration was performed with nine manually selected points

A 22.5 × 24 × 0.8 cm^3^ H.A.M. applicator (Eckert & Ziegler, Germany) was placed on the top of a 2.5 cm thick solid water phantom (Figure 3a). Three catheters spaced 2‐cm apart were inserted into the applicator in the central area. The H.A.M applicator was chosen for this study due to its simplicity in geometry and convenience for setup. The whole configuration was scanned in our CT simulator with a 1 mm slice thickness and the scan was exported to the Eclipse TPS for treatment planning. The phantom surface was covered with a grid paper (1 mm scale) and three radiopaque markers were placed on its sides. The applicator position at the time of scanning was marked on the grid paper to improve setup accuracy and reproducibility. All three catheters were digitized in Eclipse 15.6 TPS. User Origin was set as the triangulation point of the three radiopaque markers. Source positions were chosen to have a 1‐cm step size with the first source position at 2 cm and the last source position at 20 cm. The dwell time was carefully adjusted to generate a “peanut‐shape” dose distribution (Figure 3b,c). All three catheters had the same source positions and dwell times, and the dose distribution was calculated based on the TG‐43 formula in Eclipse.^27^ The “peanut‐shape” dose distribution on the phantom surface was then exported in DICOM RT format from the Eclipse TPS as the reference dose distribution.

To measure this delivered dose distribution, we exported the treatment plan to our HDR unit and delivered the plan with the same phantom setup in Figure 3a. The beam on time was adjusted according to the source strength (5.071 Ci) of the HDR unit on the day of the experiment. A 7 × 25.4 cm^2^ film piece was cut and placed under the H.A.M applicator, which can be easily identified in Figure 3a as it highlights a yellow rectangle in the central area of the grid paper. All film handling and processing followed the same protocol mentioned above in the calibration section. Two measured dose distributions––one for the 6 MV calibration and the other for the Ir‐192 calibration––were generated by converting the pixel values to doses using the corresponding calibration curve.

#### 2.5.1 Gamma analysis

The gamma analysis was performed using the RIT V6.8 patient QA analysis tool, which requires image registration between the planned and measured dose plane. In this study, manual registrations were performed by carefully aligning the centers of nine selected dwell positions––three in the top, three points in the middle, and three points in the bottom of the dose plane (Figure 3d).

Both dose planes were normalized to the max dose (global normalization). Different combinations of distance to agreement (DTA) (1, 2, 3, and 5 mm) and dose difference (1%, 2%, 3%, 4%, and 5%) were used as gamma criteria. The low‐dose threshold was kept at 10% in all analyses. Performances of the 6 MV and Ir‐192 calibrations were then evaluated with the gamma passing rate and the gamma pass/fail plot on the reference image.

#### 2.5.2 Comparison of point‐by‐point profile at low‐, medium‐, and high‐dose regions

Along each of the three catheters, the treatment plan produced a “peanut‐shape” dose distribution that had three (medium, low, and high) dose levels. This dose distribution pattern enabled us to evaluate the dose dependence of the performance of 6 MV calibration. The reference dose profile and dose difference (between the reference and measurement) profile along each catheter were calculated and exported from the RIT V6.8 Film for both 6 MV and Ir‐192 calibrations. The discrepancy, ΔR, between the two calibrations at each measurement point was calculated as:

(2) $$\begin{array}{ccc} \Delta R & = & \mathrm{abs}{\left(\frac{Meas.\;-Ref.}{Ref.}\right)}_{6MV}\\ & & \times \;100\%-\mathrm{abs}{\left(\frac{Meas.-Ref.}{Ref}\right)}_{Ir-192}\times 100\%, \end{array}$$

where Meas. is the measured dose for the corresponding calibration, and Ref is the planned dose at the measurement point. A positive value of ∆*R* means the 6 MV calibration has a larger percent error at the measurement point compared to Ir‐192 calibration, while the negative value indicates the opposite.

Statistical analysis was performed using Origin 2022 software to test if the discrepancy (ΔR) distributions are significantly different among the three dose levels for each catheter. The assumptions of normality and homogeneity of variance were tested before performing the classic *F*‐test for one‐way analysis of variance (ANOVA). If these assumptions were not met, the Kruskal–Wallis rank test, a nonparametric alternative for one‐way ANOVA *F*‐test, would be performed, followed by a post‐hoc test to determine the sources of difference. For each catheter, three pairwise comparisons were performed in the post hoc test since there were three dose levels and $C_{2}^{3}=\;3$. Boxplots of the reference dose distribution and histograms of discrepancy (ΔR) distribution in the medium‐, low‐, and high‐dose regions were generated for each catheter.

### 2.6 Uncertainty analysis

According to IAEA TECDOC No. 1585 and the American Association of Physicists in Medicine (AAPM) TG‐235, measurement uncertainty should be estimated with each influence quantity in the measurement model.^28^, ^29^ This study involved film calibration with two different dose delivery systems. Even though the same protocol was used for film handling, measurements from the two delivery systems were associated with different degrees of uncertainty.

The methodology reported by Sorriaux et al.^30^ was adopted to estimate the uncertainties in the film dosimetry system. The single film scanning reproducibility was measured by scanning three times the same film placed at the same position, and calculating the standard deviation of the readings. Similarly, the scanner homogeneity was estimated with the same film scanned at four different positions on the scanner bed around the central area. Intrasheet uniformity was quantified by measuring three equal film pieces, irradiated with the same dose, from the same sheet. They were scanned at the same position in the center of the scanner bed and the standard deviation of the three average readings was calculated. Inter‐sheet uniformity was evaluated using two sheets with the standard deviation of the two average readings from three film pieces of each sheet. The film calibration function was provided by the RIT V6.8 Film and was estimated with a typical uncertainty of 1.5% (*k* = 1).^29^

Uncertainties associated with the two treatment delivery systems were estimated with methodologies proposed in previous work.^2^, ^29^, ^31^, ^32^, ^33^ Dose calibration uncertainty of photon beam on the TrueBeam was estimated with a typical value of 0.9% for a coverage factor of one.^29^ The dose gradient of a 20 × 20 cm^2^ 6 MV beam at 10 cm depth is normally around 3.3%/cm. The film positioning uncertainty from the zeroing, MP1 traveling in the vertical direction, and Roos chamber holder titling was assumed to be ±15 mm, which translates to an uncertainty of 0.25% (*k* = 1). The photon beam profile was not perfectly flat either. The contribution to uncertainties was estimated by calculating the standard deviation of the average line dose of a 6 cm ROI placed at four off‐axis positions around the center of in‐plane and cross‐plane profiles. According to AAPM TG‐138, the uncertainty of Ir‐192 source strength was estimated to be 1.3% (*k* = 1).^31^ Moreover, Zourari et al.^32^ reported a general uncertainty of 1% for the Acuros BrachyVision dose calculation algorithm. Since the coverage factor was not quoted, we assumed *k* = 2. The contribution of dwell position on dose delivery was estimated to be 2% (*k* = 2) based on a study from Palmer et al.^33^ Unlike the 6 MV photon beam, the Ir‐192 source has a very sharp dose fall‐off. The thickness of the polyester layer of EBT3 film is approximately 0.1 mm, which inherently contributes to the uncertainty in determining the distance between the film active layer and Ir‐192 source center. A positioning error of 0.1 mm is estimated to have an uncertainty of 1.3% (*k* = 1) in dose measurement, considering the dose gradient of Ir‐192 at 2 cm is 13.2% per mm from TPS.^2^, ^29^

## 3 RESULTS

### 3.1 Calibration curves

**FIGURE 4:**
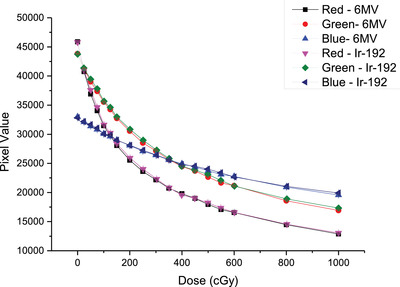
EBT3 film calibration curves (pixel value vs. dose) for 6 MV and Ir‐192 calibrations. Error bars (*k* = 1) are smaller than data points

Figure 4 presents the calibration curves (pixel value vs. dose) for the red, green, and blue channels. The Ir‐192 calibration curve shows slightly under‐response. That is, at the same dose level, films irradiated with the Ir‐192 source had larger pixel values (or less film darkness), compared to that exposed to the 6 MV photon beam. The average difference in pixel value (for the same dose) between the 6 MV and Ir‐192 calibration curves was found to be < 1% for the red and green channels, and < 0.5% for the blue channel.

### 3.2 Single‐dose comparison

**FIGURE 5:**
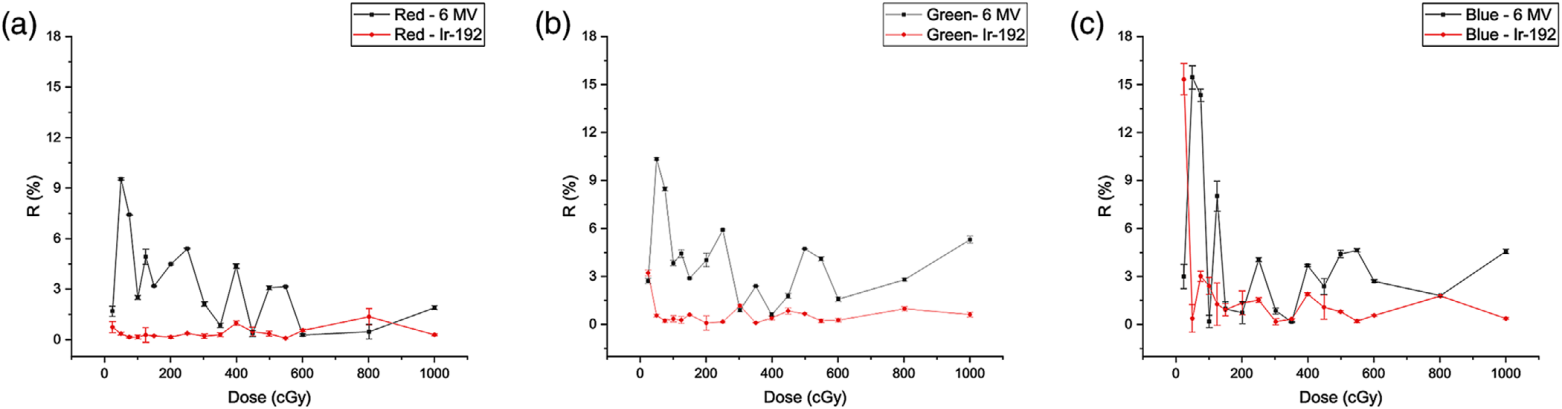
Percent error from the reference dose, $R=abs(\frac{{\bar{D}}_{ROI}-D_{ref}}{D_{ref}})\times 100\%$ in Equation (1), where ${\bar{D}}_{ROI}$ was converted from the pixel value using the 6‐MV or Ir‐192 calibration curves for (a) red, (b) green, and (c) blue channels, with a coverage factor of *k* = 1

The accuracy of 6 MV and Ir‐192 calibrations for determining a single dose delivered with the HDR afterloader is shown in Figure 5. The (mean ± standard deviation) percent error of $R\;=\;\text{abs}(\frac{{\bar{D}}_{ROI}-D_{ref}}{D_{ref}})\;\times \;100\%$ as defined in Equation (1) is generally small for the Ir‐192 calibration between 0 and 1000 cGy, particularly for the red ($\bar{R}=\;0.40\pm 0.09\%$) and green ($\bar{R}=\;0.63\pm 0.07\%$) channels. For the 6 MV calibration, the (mean ± standard deviation) percent errors were 3.27±0.08%, 3.93±0.07%, and 4.23±0.19%, respectively, for the red, green, and blue channels across the whole dose range, and the red channel had the best performance. The better performance of Ir‐192 calibration could be explained by the use of the same film for verification. To mitigate its effect, we have minimized the overlapped pixels between ROI_1_ in calibration and ROI_2_ in verification. A closer look at the individual performance of the 6 MV and Ir‐192 calibrations in the red channel shows the mean percent errors (0.87% vs. 0.72%) were comparable for both calibrations when the dose is larger than 600 cGy. However, when the dose is less than 250 cGy, the Ir‐192 calibration kept a similar performance ($\bar{R}=\;0.30\pm 0.13\%$), while the 6 MV calibration had a significantly worse performance ($\bar{R}=\;4.89\pm 0.12\%$).

### 3.3 Comparison of 2D dose distribution

Since the red channel had the best overall performance, the analyses and comparison of 2D dose distribution were all based on the red channel data only.

#### 3.3.1 Gamma analysis

Table 1 shows the passing rates with various gamma criteria. Under 1 mm/1% and 1 mm/2%, the Ir‐192 calibration had a passing rate of 62.4% and 91.9%, respectively, while the 6 MV calibration only had 40.3% and 83.5%. It was also observed that at all levels of DTA limit, both 6 MV and Ir‐192 calibrations resulted in a passing rate better than 94% if the dose difference criterion was set to be 3% or larger.

**TABLE 1:** Gamma analysis results using different DTA (mm)/dose diff. (%) criteria with global normalization for Ir‐192 and 6 MV calibrations

	**DTA (mm)**							
	**1**		**2**		**3**		**4**	
**Dose diff. (%)**	**6 MV**	**Ir‐192**	**6 MV**	**Ir‐192**	**6 MV**	**Ir‐192**	**6 MV**	**Ir‐192**
1	40.3	62.4	67.4	85.7	79.3	92.3	88.0	95.6
2	83.5	91.9	96.4	98.9	98.0	99.5	98.8	99.8
3	94.5	95.8	99.6	99.5	99.9	99.8	99.9	99.9
4	96.9	97.2	99.8	99.7	99.9	99.9	99.9	99.9
5	98.3	98.0	99.8	99.8	99.9	99.9	100.0	99.9

**FIGURE 6:**
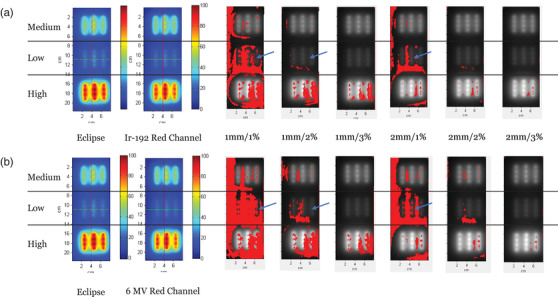
Gamma pass/fail plot for (a) Ir‐192 calibration and (b) 6 MV calibration. All points with the gamma index larger than 1 are shown in red. The average dose of the medium‐, low‐, and high‐dose regions for the center catheter from Eclipse TPS is 385.9±22.4 cGy, 191.2±8.4 cGy, and 691.5±29.7 cGy

To evaluate the pattern of failure for the gamma analysis, we generated the gamma pass/fail plot on the reference dose plane (Figure 6) using the RIT V6.8 Film. In Figure 6, all points with the gamma index larger than 1 are shown in red. Obviously, the 6 MV calibration had more failing points no matter which gamma criterion we chose. Moreover, by comparing the gamma fail/pass plots, we found that the 6 MV calibration caused significantly more failing points in the middle area of the dose plane, especially for the 1 mm/1% 1 mm/2%, 2 mm/1%, and 2 mm/2% criteria. Since the middle area corresponds to the low‐dose region in Figure 6, this result is consistent with the prior finding in single‐dose comparison that the 6 MV calibration had a larger error for low‐dose measurements.

#### 3.3.2 Comparison in low‐, medium‐, and high‐dose regions

**FIGURE 7:**
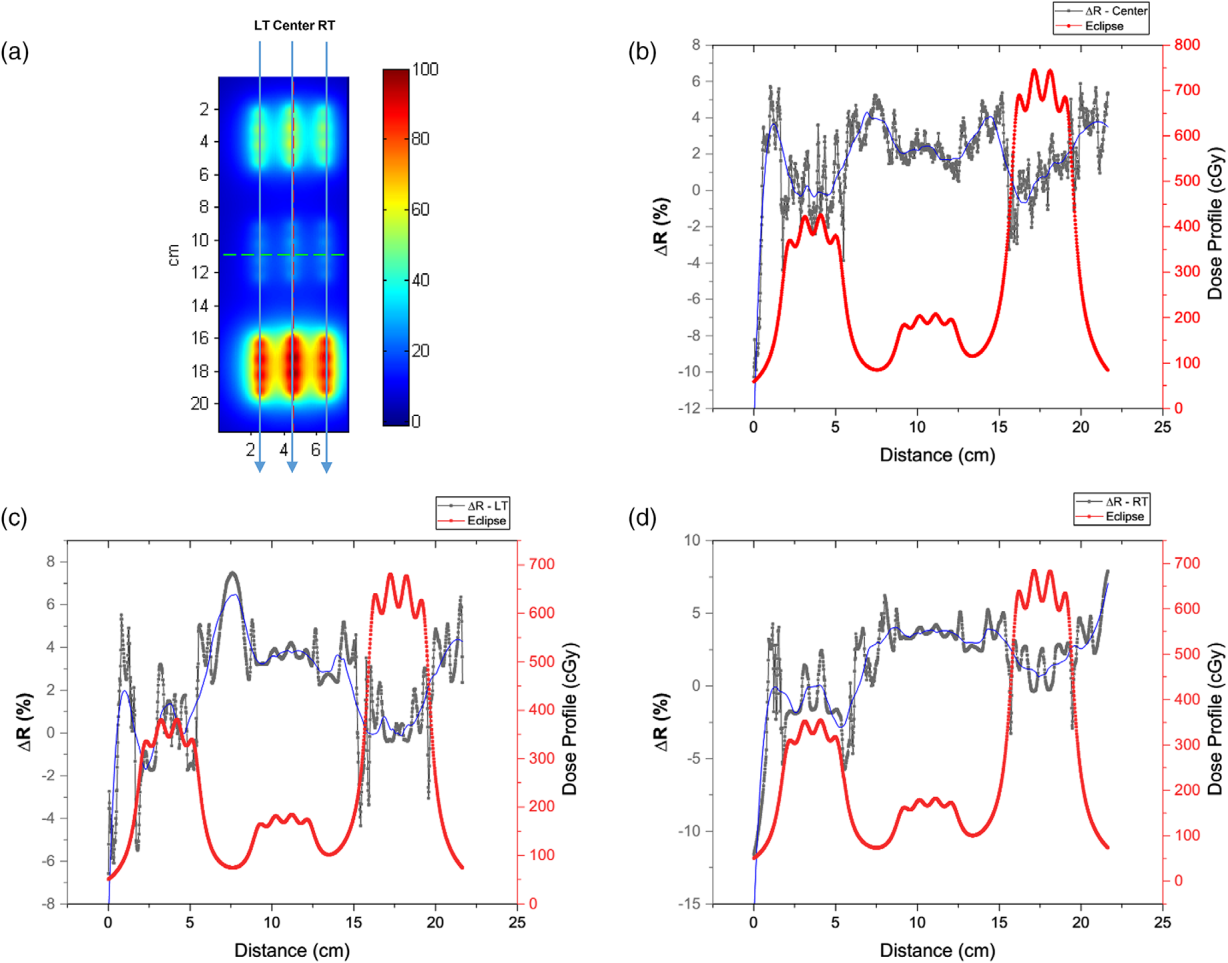
(a) Illustration of profiles drawn on the dose plane; the discrepancy, $\Delta R=abs{\left(\frac{Meas.\;-Ref.}{Ref.}\right)}_{6MV}\times 100\%-abs{\left(\frac{Meas.-Ref.}{Ref}\right)}_{Ir-192}\times 100\%$ is calculated and plotted together with the dose profile in red for (b) center, (c) left, and (d) right. The blue curves in (b), (c), and (d) are the smoothed results of ∆*R*

To further investigate the performance of the 6 MV calibration at different dose levels, we calculated the discrepancy $\Delta R\;=\mathrm{abs}{\left(\frac{Meas.\;-Ref.}{Ref.}\right)}_{6MV}\times 100\%-\mathrm{abs}{\left(\frac{Meas.-Ref.}{Ref}\right)}_{Ir-192}\times 100\%$ defined in Equation (2) along the tracks of the three (left, center, and right) catheters drawn as the blue downward arrow lines on the dose plane in Figure 7a.

**FIGURE 8:**
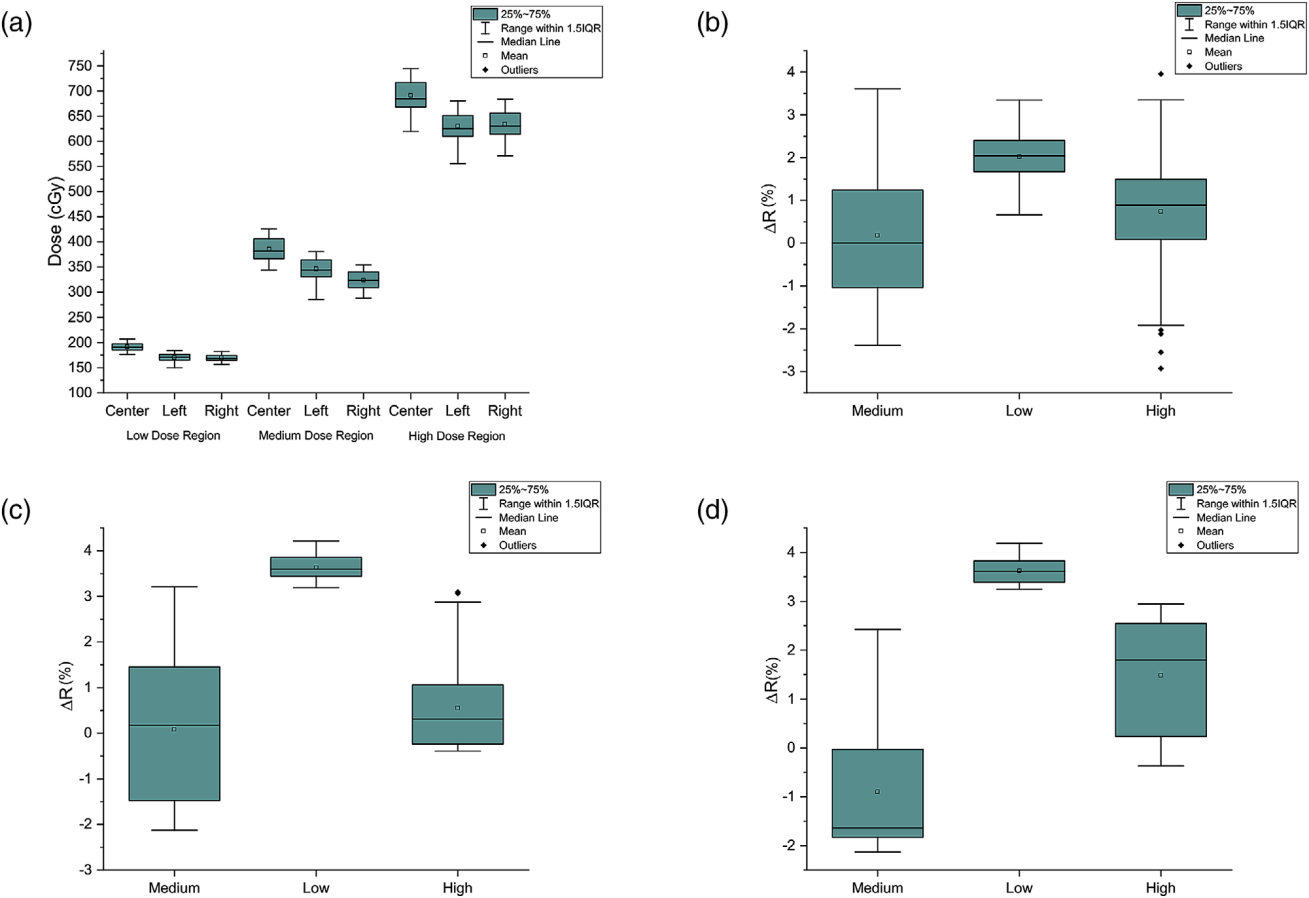
Boxplots of (a) reference dose distribution in medium‐, low‐, and high‐dose regions along left, center, and right dose planes, and (b–d) discrepancy, ΔR, distribution in three dose regions for (b) center, (c) left, and (d) right catheters

Figure 7b–d plots the discrepancy *∆R* for the (b) center, (c) left, and (d) right catheters, with the blue curves in each figure being the smoothed results of ∆*R*. The medium‐, low‐, and high‐dose regions were defined as the line of interest (LOI) from 2.00 to 5.25 cm, 9.04 to 12.29 cm, and 16.04 to 19.29 cm, respectively, along the x‐axis in Figure 7b–d. All analyses were performed within these three LOIs. Figure 8a presents the boxplots of the planned dose distribution in the medium‐, low‐, and high‐dose regions. Table 2 summarizes the (mean ± standard deviation) discrepancy, ΔR, between the Ir‐192 and 6 MV calibrations in the three dose regions along the center, left, and right catheters. If we assume that the Ir‐192 calibration is the gold standard, a larger discrepancy, ΔR, indicates a more significant difference between these two calibrations, and, therefore, less accurate for the 6 MV calibration.

**TABLE 2:** Summary (mean ± standard deviation) of the average dose and discrepancy, ΔR, between the Ir‐192 and 6 MV calibrations in the medium‐, low‐, and high‐dose regions along the center, left, and right catheters (k = 1)

		**Average dose (cGy)**	$\bar{\Delta R}$ **(%)**
Center	Medium	385.89 ± 22.38	0.18 ± 1.40
	Low	191.20 ± 8.41	2.03 ± 0.53
	High	691.50 ± 29.65	0.74 ± 1.13
Left	Medium	346.45 ± 20.72	0.08 ± 1.56
	Low	170.60 ± 7.56	3.63 ± 0.29
	High	629.89 ± 27.62	0.55 ± 0.89
Right	Medium	324.45 ± 17.66	−0.90 ± 1.34
	Low	168.98 ± 7.08	3.63 ± 0.26
	High	634.20 ± 27.07	1.48 ± 1.17

**FIGURE 9:**
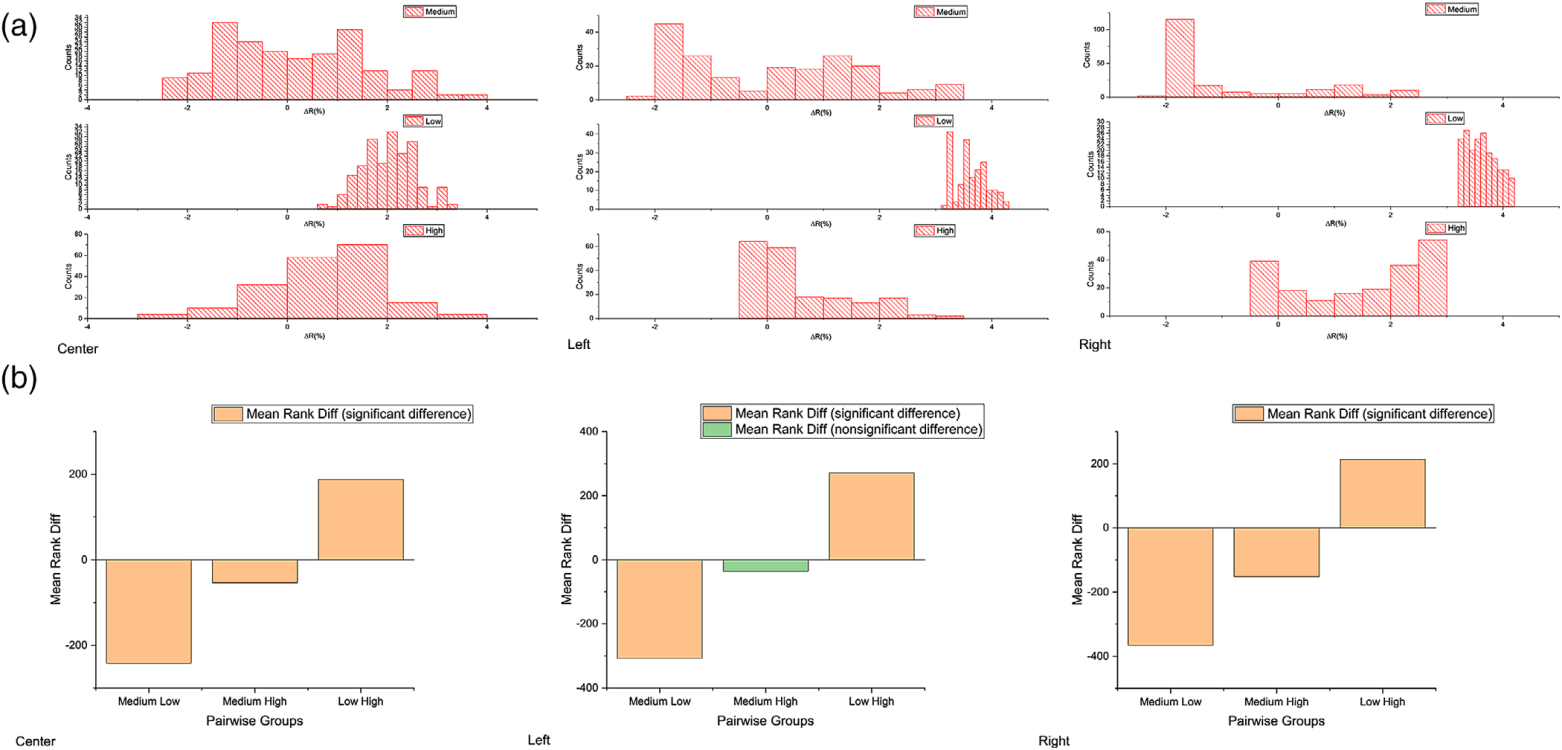
(a) Histograms of discrepancy, ΔR, distribution in the three‐dose regions, and (b) multiple paired comparison results from Dunn's test, for the center, left, and right dose profiles

Shapiro–Wilk test was used to evaluate the normality of the discrepancy (∆*R*) distribution in the medium‐, low‐, and high‐dose regions from each catheter. Only data in the low‐dose region of the center catheter demonstrated to be significantly drawn from a normally distributed population at the 0.05 significance level, while the rest all rejected the normality at the same significance level. Homogeneity of variance among the three data samples from each catheter was tested with Levene's test. Test results indicated the population variances are significantly different in the medium‐, low‐, and high‐dose regions at the 0.05 significance level. Figure 9a shows the histograms of the discrepancy (∆*R*) distribution in the three‐dose regions for each catheter.

Since both normality and homogeneity of variance could not be assumed, the Kruskal–Wallis rank test was performed, followed by the Dunn's test as the post hoc test. The Kruskal–Wallis rank test rejected the null hypothesis at the 0.05 significance level, indicating at least two of the three data for each catheter are significantly different in terms of mean rank (Table 3). Dunn's pairwise comparison tests in Figure 9b further demonstrated that the mean ranks in medium‐, low‐, and high‐dose regions are significantly different from each other at the 0.05 significance level for the center and right catheters, except for the left catheter, where the mean ranks in medium‐ and high‐dose region are not significantly different. Table 3 presents the mean ranks and median of ∆*R* in the three‐dose regions for each catheter. The low‐dose region had the highest mean rank for the center, left, and right catheters, indicating that the 6 MV calibration has the worst performance in this dose region.

**TABLE 3:** Descriptive statistics and ranks of a discrepancy, ΔR, in the medium‐, low‐, and high‐dose regions for the center, left, and right catheters

		**Descriptive statistics**					**Ranks**		**Kruskal–Wallis test**		
		**Min**	**Q1**	**Median**	**Q3**	**Max**	**Mean**	**Sum**	**H‐value**	**DF**	***p*‐value**
Center	Medium	–2.39	–1.04	0.00	1.25	3.61	191.35	36 931	222.44	2	5.0E‐49
	Low	0.66	1.67	2.04	2.40	3.35	433.28	83 624			
	High	–2.93	0.07	0.89	1.50	3.96	245.36	47 355			
Left	Medium	–2.12	–1.49	0.17	1.46	3.21	175.33	33 838	389.72	2	2.4E‐85
	Low	3.19	3.43	3.59	3.86	4.22	482.95	93 210			
	High	–0.39	–0.25	0.31	1.10	3.10	211.72	40 862			
Right	Medium	–2.13	–1.83	–1.64	–0.02	2.42	117.40	22 658	465.22	2	9.5E‐102
	Low	3.24	3.39	3.61	3.83	4.19	483.00	93 219			
	High	–0.37	0.23	1.80	2.55	2.94	269.60	52 033			

Note: The last column shows the test results of the Kruskal–Wallis rank test with a 0.05 significance level.

Abbreviations: DF, degree of freedom; H, Kruskal–Wallis test statistics; Max, maximal value; Min, minimal value; Q1, first quartile; Q3, third quartile.

### 3.4 Uncertainty budget

The uncertainty budgets were estimated separately and are shown in Table 4. The difference in the expanded uncertainty (*k* = 2) reflects mainly the dose delivery accuracy caused by their mechanical design and beam characteristics.

**TABLE 4:** Uncertainty budgets for the Ir‐192 and 6 MV calibrations

		**Ir‐192 calibration**		**6 MV calibration**	
**Category**	**Source of uncertainty**	**Type A**	**Type B**	**Type A**	**Type B**
Treatment delivery	Linac calibration for reference exposure				0.9
	Beam uniformity				0.02
	Source strength	1.3			
	Treatment planning system calculation		0.5		
	Source dwell position		1		
	Film position in setup	1.3		0.25	
Film dosimetry	Single film scanning reproducibility	0.14		0.14	
	Intrasheet uniformity	0.16		0.16	
	Intersheet uniformity	0.33		0.33	
	Film calibration fit functions		1.5		1.5
	Scanner homogeneity		0.96		0.96
	Total uncertainty (*k* = 1)	2.83		2.05	
	Expanded uncertainty (*k* = 2)	5.66		4.10	

Note: Data were extracted from the red channel of scanned images of the irradiated EBT3 films.

## 4 DISCUSSION

The overall energy dependence of radiochromic film is composed of two components, intrinsic energy dependence and extrinsic energy dependence (or the absorbed‐dose energy dependence). The intrinsic energy dependence relates the chemical changes in film to the absorbed dose in film, while the other connects the dose to water with the dose to film. This study focused on the dosimetric impact of the overall energy dependence of EBT3 film, instead of calculating its individual components. The main purpose was to evaluate the accuracy and feasibility of 6 MV calibration for dose measurement of an Ir‐192 source.

As shown in Figure 4, the Ir‐192 calibration curve is slightly above the 6 MV calibration curve for all three channels, indicating that the EBT3 film has a lower response (or less darkness) when exposed to the Ir‐192 source for the same dose level compared to the 6 MV photon beam. This can be explained by the decreased efficiency of film polymerization as energy decreases.^22^ Among the three color channels, the blue channel showed the least under‐response (0.5%). This is not because the blue channel is less energy‐dependent, but due to the presence of the additive yellow marker dye in the active layer of the film. Most of the blue light is absorbed by the yellow marker dye so the difference in the absorption of blue lights due to the chemical change of film color became relatively minor in comparison to other colors. Results from the single‐dose comparison (Figure 5) showed a similar trend that the difference between these two calibrations is least significant for the blue channel. Moreover, due to the loss of signal to the yellow dye, the blue channel in fact had the largest variation of percent error in both calibrations. Our findings of the channel effect on film dose measurements agree with prior publications that the red channel has superior performance than the other two channels in the dose range up to 10 Gy.^13^

In the present work, we observed that the dosimetric accuracy of 6 MV calibration for the EBT3 film depends on the dose levels. For doses less than 250 cGy, we found a larger mean deviation (4.89%) from the reference in the single‐dose comparison using the 6 MV calibration, while it is only 0.87% for doses larger than 600 cGy (Figure 5). The evaluation of 2D dose distribution based on gamma pass/fail plots (Figure 6) also showed that there were more failing points in the low‐dose region (<200 cGy) under all gamma criteria with global normalization.

This finding was further validated in the point‐by‐point dose profile comparison (Figures 7, 8, 9). We found that the mean discrepancies, $\bar{\Delta R}$, between the 6 MV and Ir‐192 calibrations are much higher in the low‐dose region than in the medium‐ and high‐dose regions (Table 2) for all three dose profiles, indicating a worse performance for the 6 MV calibration in these low‐dose regions. In contrast, for medium‐ and high‐dose regions, the mean discrepancy, $\bar{\Delta R}$, is close to zero with a larger uncertainty, which indicates the 6 MV and Ir‐192 calibration are commensurate in those dose regions. To be mentioned, the right catheter seems to have a larger value of $\bar{\Delta R}$ in the medium‐ and high‐dose regions than the left catheter. But considering the associated uncertainties, these two profiles are still comparable.

Moreover, results from the Kruskal–Wallis rank test and the Dunn's multiple comparison test demonstrated the dose dependence of the discrepancies, ΔR, between the 6 MV and Ir‐192 calibrations, particularly for the low (<200 cGy) dose region. As shown in Figure 9b, the difference in mean rank was statistically significant between the low and the other two (medium and high) dose regions for all three catheters. The difference between the medium‐ and high‐dose regions, on the other hand, was much smaller and was not statistically significant only for the left catheter. These results are consistent with an early report by Richter et al.^34^ who found that variation in energy response for the EBT film is related to the dose level––higher doses have a smaller energy dependency than lower doses (up to 25% between 0.5 and 10 Gy). Massillon et al.^20^ also published that a larger variation (up to 11%) in energy response was observed for the EBT3 film at doses less than 2 Gy.

In this study, we adopted a globally normalized gamma evaluation even though Palmer et al.^35^ suggested the local normalization should be used to improve the accuracy in low‐dose regions. Investigation of the optimal analysis tool for brachytherapy dose evaluation is beyond the scope of this study. Readers are referred to the AAPM TG 218 report for more discussions on the efficacy of the gamma index.^15^ We believe the choice of normalization point would have a minimal effect on our conclusions because we focused on the comparison of two calibrations under the same criteria. That is, the additional inaccuracy due to different normalization schemes, if there was any, would appear in both calibrations, and be canceled out when calculating the dose difference between these two calibrations. This was evidenced by the fact that consistent results were found in the single‐point dose‐comparison and point‐by‐point profile comparison of this study. Given that the 1 mm/2% gamma criterion with global normalization shows an approximately 10% difference in passing rate between the two calibrations, we do not expect the conclusions would be reversed with local normalization.

Overall, the film processing protocol adopted in this study was shown to be reliable with an expanded uncertainty of 3.64% (*k* = 2). The detailed uncertainty budget analysis for EBT3 film dosimetry with the two (linac and HDR) dose delivery systems includes uncertainties from linac does calibration, beam uniformity, source strength, TPS dose calibration, dwell position, scanner uniformity, and so on. The linac‐based system was estimated to have a smaller expanded uncertainty (*k* = 2) of 4.1%, compared to 5.66% (*k* = 2) of the Ir‐192‐based HDR delivery system. Both were considered reasonable. To be mentioned, the inter‐sheet uniformity was found to be 0.33%, which is better than that of EBT2 film (1.6% by Richley et al.^36^ and < 1% by Mizuno et al.^37^). The single film scanning reproducibility was only estimated at a single dose level (200 cGy) because Sorriaux et al.^30^ showed that the dose dependence of single film scanning reproducibility is negligible on EBT3 film among 0.4, 2, and 6.5 Gy. The superior performance of EBT3 film is probably due to the use of double layers of polyester around the active layer, which increases the homogeneity and reduces the artifacts, such as Newton's ring.^29^

Other sources of uncertainty, such as humidity or dose‐rate effect, are considered negligible. Leon‐Marroquin et al.^38^ reported that when the film is immersed in water for a very long time, the higher humidity might increase the film response. This was not a concern in this study as the treatment time was much shorter than that required for significant water penetration. In addition, all films were stored in the same storage room with temperature control. The humidity was thus believed to have minimal effect on the dose measurement. Calibration and verification measurements with the Ir‐192 source were performed at a source strength of 5.071 and 5.958 Ci, respectively. This change in activity could result in an 18% difference in dose rate. According to the vendor's specifications,^39^ the difference in net optical density is less than 5% for a dose rate range of two orders of magnitude (between 3.4 and 0.034 Gy/min). Therefore, the effect of 18% dose rate change on this study was negligible.

As mentioned in the Introduction, EBT3 film is a popular choice for validating dose distribution of the 3D‐printed applicators.^18^, ^19^ Results from this study indicate that the accuracy of film dosimetry using the 6 MV calibration could be improved if measurements of the low‐dose (<250 cGy) regions could be avoided. This can potentially be achieved by scaling up the dose linearly. Alternatively, we can recalibrate the film directly using the Ir‐192 source, when validating the dose distribution of an HDR brachytherapy plan with 3D‐printed applicators, especially for gynecologic cancer patients who could receive 2.5 Gy per fraction.^40^

## 5 CONCLUSIONS

To the best of our knowledge, this is the first work that evaluated the dosimetric accuracy of 6 MV calibration on EBT3 film for Ir‐192 HDR brachytherapy in the dose range up to 1000 cGy. We have developed a feasible calibration protocol for direct calibration of the ETB3 film in water with the Ir‐192 HDR source and used this protocol as the gold standard to evaluate the dosimetric accuracy of 6 MV calibration. The uncertainty budget analysis showed that the uncertainty is reasonable for both calibrations. Direct calibration using the Ir‐192 source has an expanded uncertainty of 5.66%, compared to 4.1% using the 6 MV photon beam.

Performance associated with the two (6 MV and Ir‐192) calibrations was analyzed by comparing the planned dose distributions of an HDR brachytherapy plan with those measured using EBT3 films calibrated with the two sources. The dosimetric accuracy of 6 MV calibration was found to be inferior only in the low‐dose region, for example, below 250 cGy, compared to the direct Ir‐192 calibration. Therefore, we concluded that the 6 MV calibration is clinically acceptable, considering the overall uncertainties. The accuracy in low‐dose (<250 cGy) regions can be improved by scaling up the dose to around 600 cGy for the 6 MV calibration. When dose scaling is not available, the direct Ir‐192 calibration should be performed.

## AUTHOR CONTRIBUTIONS

Lyu Huang was responsible for research design, literature review, data collection, data analysis and manuscript writing. Hani Gaballa was responsible for research design and data collection. Jenghwa Chang was responsible for research design, data analysis, and manuscript editing.

## CONFLICT OF INTEREST

The authors declare that they have no conflict of interest.
